# Draft genome sequence of *Nocardia* sp. strain JMUB6875 isolated from a patient with disseminated nocardiosis

**DOI:** 10.1128/mra.00735-24

**Published:** 2024-10-02

**Authors:** Takayuki Suzuki, Shinya Watanabe, Nayuta Seto, Momori Honjo, Longzhu Cui, Shuji Hatakeyama

**Affiliations:** ^1^Division of Infectious Diseases, Department of Infection and Immunity, Jichi Medical University, Tochigi, Japan; 2 Division of Bacteriology, Department of Infection and Immunity, Jichi Medical University, Tochigi, Japan; 3 Division of General Medicine, Jichi Medical University Saitama Medical Center, Saitama, Japan; 4 Trauma and Emergency Center, Fukaya Red Cross Hospital, Saitama, Japan; 5 Division of General Medicine, Center for Community Medicine, Jichi Medical University, Tochigi, Japan; Department of Biological Sciences, Wellesley College, Wellesley, Massachusetts, USA

**Keywords:** *Nocardia*

## Abstract

We report the draft genome sequence of the *Nocardia* sp. strain JMUB6875, isolated from a blood culture specimen of a patient with disseminated nocardiosis. This strain appears to be a potentially novel species closely related to *Nocardia concava*.

## ANNOUNCEMENT

*Nocardia* species are aerobic, gram-positive, weakly acid-fast bacteria that cause localized or systemic infections in humans (1). Currently, 130 validly published species belong to the genus *Nocardia* (https://lpsn.dsmz.de/genus/nocardia). Although accurate species identification and antimicrobial susceptibility results are critical for clinicians treating nocardiosis, identification is difficult for some *Nocardia* species, even with 16S ribosomal RNA (rRNA) sequencing (2). We report the draft genome sequence of a novel *Nocardia* sp. strain JMUB6875, isolated from a blood culture specimen of a Japanese patient with disseminated nocardiosis in 2022. This study adhered to the guidelines of the Declaration of Helsinki.

*Nocardia* sp. strain JMUB6875 was detected after 1 day of culturing the patient’s blood in BD BACTEC Plus aerobic medium (Becton, Dickinson, and Company, Franklin Lakes, NJ, USA) and subcultured on sheep blood agar at 37°C for 3 days. Genomic DNA was extracted using the QIAamp DNA Mini Kit (Qiagen, Hilden, Germany). The 16S rRNA sequence was amplified using KOD One PCR Master Mix (Toyobo, Tokyo, Japan) and universal primers 27F (AGAGTTTGATCMTGGCTCAG) (3) and 1492R (GGTTACCTTGTTACGACTT) (4). Thermal cycling included an initial denaturation step at 98°C for 3 minutes, followed by 34 cycles of 98°C for 10 seconds, 60°C for 5 seconds, and 68°C for 10 seconds. Sanger sequencing (Eurofins Genomics, Tokyo, Japan) revealed that the 16S rRNA sequence of this strain (LC819931.1) had 99.29% identity with *Nocardia yunnanensis* strain CFH S0054^T^ (CP032568.1) and 98.50% identity with *Nocardia concava* strain DSM 44804^T^ (NR_115958.1). However, this strain could not be identified as *N. yunnanensis* according to CLSI MM18-A guidelines, which require at least 99.6% homology for species-level identification of *Nocardia* spp (5). Paired-end libraries were prepared from genomic DNA using the Nextera XT Library Prep (Illumina, Inc., San Diego, CA, USA). Sequencing was performed using an Illumina HiSeq XTen system (2 × 150 bp) to generate 2,006,710 paired-end reads. Raw reads were trimmed and filtered using fastp v0.23.4 (6), yielding 1,829,848 reads. Default parameters were used unless otherwise noted. The genome sequence was assembled using Shovill v1.1.0 (7). Draft genome sequences were annotated using the DDBJ Fast Annotation and Submission Tool (DFAST) v1.2.20 (8). The assembly generated 439 contigs with an *N*_50_ value of 38,659 bp, a maximum contig size of 145,397 bp, a draft genome size of 8,335,144 bp, an average genome coverage of 36×, and an average GC content of 67.9%. The annotated genome contained 7,659 coding sequences, five rRNAs, 76 tRNAs, and zero clustered regularly interspaced short palindromic repeats. We also analyzed the *gyrB* sequence, effective for refining taxonomic classification (9), using Clustal Omega v1.2.4. The 1,200-bp *gyrB* sequence showed 97% and 94% identity to *N. concava* (AB450778.1) and *N. yunnanensis* (NZ_CP032568.1), respectively. Furthermore, average nucleotide identity (ANI) based on BLAST +comparison (ANIb) was calculated using JspeciesWS v4.1.1 (10). *Nocardia* sp. strain JMUB6875 had ANIb values of 92.61% and 84.82% with *N. concava* NBRC 100430^T^ and *N. yunnanensis* CFH S0054^T^, respectively.

In conclusion, *Nocardia* sp. strain JMUB6875 is a potentially novel species closely related to *N. concava*. This draft genome will improve the understanding of *Nocardia* taxonomy.

## Data Availability

The whole-genome data from this study were deposited in DDBJ/ENA/GenBank under the accession number BAAFGR000000000.1, the first version of the master record for the whole-genome shotgun project, and can be accessed via the WGS section of this record. The raw data were deposited in the SRA under the accession number DRR535498. The BioSample and BioProject accession numbers are SAMD00754404 and PRJDB17663, respectively. The 16S rRNA sequence as determined by Sanger sequencing was deposited under the accession number LC819931.1.

## References

[1] Traxler RM, Bell ME, Lasker B, Headd B, Shieh W-J, McQuiston JR. 2022. Updated review on Nocardia species: 2006-2021. Clin Microbiol Rev 35:e0002721. doi:10.1128/cmr.00027-21 36314911 PMC9769612

[2] Roth A, Andrees S, Kroppenstedt RM, Harmsen D, Mauch H. 2003. Phylogeny of the genus Nocardia based on reassessed 16S rRNA gene sequences reveals underspeciation and division of strains classified as Nocardia asteroides into three established species and two unnamed taxons. J Clin Microbiol 41:851–856. doi:10.1128/JCM.41.2.851-856.2003 12574299 PMC149683

[3] Lane DJ. 1991. 16S/23S rRNA sequencing, p 115–175. In Stackebrandt E, Goodfellow M (ed), Nucleic acid techniques in bacterial systematics. John Wiley and Sons, New York, NY.

[4] Turner S, Pryer KM, Miao VP, Palmer JD. 1999. Investigating deep phylogenetic relationships among cyanobacteria and plastids by small subunit rRNA sequence analysis. J Eukaryot Microbiol 46:327–338. doi:10.1111/j.1550-7408.1999.tb04612.x 10461381

[5] Clinical and Laboratory Standards Institute. 2008. Interpretive criteria for identification of bacteria and fungi by DNA target sequencing. Approved guideline, CLSI document MM18-A. Clinical and Laboratory Standards Institute, Wayne, PA.

[6] Chen S. 2023. Ultrafast one-pass FASTQ data preprocessing, quality control, and deduplication using fastp. Imeta 2:e107. doi:10.1002/imt2.107 38868435 PMC10989850

[7] Seemann T. 2020. Shovill: assemble bacterial isolate genomes from Illumina paired-end reads. Github. https://github.com/tseemann/shovill.

[8] Tanizawa Y, Fujisawa T, Nakamura Y. 2018. DFAST: a flexible prokaryotic genome annotation pipeline for faster genome publication. Bioinformatics 34:1037–1039. doi:10.1093/bioinformatics/btx713 29106469 PMC5860143

[9] Takeda K, Kang Y, Yazawa K, Gonoi T, Mikami Y. 2010. Phylogenetic studies of Nocardia species based on gyrB gene analyses. J Med Microbiol 59:165–171. doi:10.1099/jmm.0.011346-0 19833784

[10] Richter M, Rosselló-Móra R, Oliver Glöckner F, Peplies J. 2016. JSpeciesWS: a web server for prokaryotic species circumscription based on pairwise genome comparison. Bioinformatics 32:929–931. doi:10.1093/bioinformatics/btv681 26576653 PMC5939971

